# Decreased STEC shedding by cattle following passive and active vaccination based on recombinant *Escherichia coli* Shiga toxoids

**DOI:** 10.1186/s13567-018-0523-0

**Published:** 2018-03-07

**Authors:** Nadine Schmidt, Stefanie A. Barth, Jana Frahm, Ulrich Meyer, Sven Dänicke, Lutz Geue, Christian Menge

**Affiliations:** 1 Friedrich-Loeffler-Institut (FLI)/Federal Research Institute for Animal Health, Institute of Molecular Pathogenesis, Jena, Germany; 2 Friedrich-Loeffler-Institut (FLI), Institute of Animal Nutrition, Brunswick, Germany; 3 Justus Liebig Universität Giessen, Institute for Hygiene and Infectious Diseases of Animals, Giessen, Germany

## Abstract

The principal virulence factor of Shiga toxin (Stx)-producing *Escherichia coli* (STEC), the eponymous Stx, modulates cellular immune responses in cattle, the primary STEC reservoir. We examined whether immunization with genetically inactivated recombinant Shiga toxoids (rStx1_MUT_/rStx2_MUT_) influences STEC shedding in a calf cohort. A group of 24 calves was passively (colostrum from immunized cows) and actively (intra-muscularly at 5^th^ and 8^th^ week) vaccinated. Twenty-four calves served as unvaccinated controls (fed with low anti-Stx colostrum, placebo injected). Each group was divided according to the vitamin E concentration they received by milk replacer (moderate and high supplemented). The effective transfer of Stx-neutralizing antibodies from dams to calves via colostrum was confirmed by Vero cell assay. Serum antibody titers in calves differed significantly between the vaccinated and the control group until the 16^th^ week of life. Using the expression of activation marker CD25 on CD4^+^CD45RO^+^ cells and CD8α^hi^CD45RO^+^ cells as flow cytometry based read-out, cells from vaccinated animals responded more pronounced than those of control calves to lysates of STEC and *E. coli* strains isolated from the farm as well as to rStx2_MUT_ in the 16^th^ week. Summarized for the entire observation period, less fecal samples from vaccinated calves were *stx*_1_ and/or *stx*_2_ positive than samples from control animals when calves were fed a moderate amount of vitamin E. This study provides first evidence, that transfer to and induction in young calves of Stx-neutralizing antibodies by Shiga toxoid vaccination offers the opportunity to reduce the incidence of *stx*-positive fecal samples in a calf cohort.

**Electronic supplementary material:**

The online version of this article (10.1186/s13567-018-0523-0) contains supplementary material, which is available to authorized users.

## Introduction

Enterohemorrhagic *Escherichia coli* (EHEC), a subset of Shiga toxin-producing *E. coli* (STEC), is a food-borne pathogen that can evoke life-threatening diseases in humans such as hemorrhagic colitis and hemolytic-uremic syndrome. EHEC serotype O157:H7/H− is implicated in most EHEC outbreaks worldwide. However, human infections by non-O157:H7 serotypes (e.g. O91, O26, O113) frequently occur in Germany and other European countries [1]. The only virulence marker common to all STEC serotypes is the possession of a gene encoding for Shiga toxin (Stx). Calves get infected orally with a plethora of different STEC strains present in their environment early in life, but rarely develop clinical signs of infection. Many STEC strains are able to colonize the bovine intestine [2–6], including non-O157:H7 [7–9]. Cattle may shed these bacteria for several month in quantities that may be considerably high at some sampling points [10–13] making cattle, besides other ruminants, an important reservoir for STEC strains associated with human diseases. To reduce the risk of STEC entering the food chain, interventions must be applied at several stages starting at cow and herd level and continuing in slaughterhouses, processing plants, distributors, and households [14, 15].

Even though previous attempts to develop vaccination strategies in cattle were promising they only partially reduce STEC excretion and the effect was mostly restricted to single subpopulations of STEC, e.g. O157 strains [14, 16]. Long-term studies on anti-Stx antibody titers in serum and fecal STEC shedding by cattle unveiled significantly delayed humoral immune responses following experimental STEC infection [17] and natural exposure [12]. Delayed adaptive cellular immune responses was also shown after experimental STEC infection [17]. The principal STEC virulence factor, the eponymous Stx, modulates cellular immune responses in cattle [18–21]. In vitro and in vivo studies revealed that Stx operates during the early phases of immune activation rather than depressing an established immunity [17, 20, 22, 23]. Current knowledge of STEC shedding dynamics and influences of Stx on immune responses suggests, that Stx may hinder the development of an effective immune response by hitherto immunologically naïve animals upon first STEC contact at early calves’ ages.

Inactivation of Stx by genetic modification located within the enzymatically active cleft of Stx resulted in toxoids (rStx1_MUT_ and rStx2_MUT_) with retained antigenicity and immunogenicity but lost immunomodulatory properties in cattle [24]. Immunisation of sows with Stx2e toxoid [25, 26] was shown to trigger maternal immunity which protects offspring against edema disease [27] and fully protected the animals when challenged with native Stx2e [28]. Induction of humoral and cellular immune responses by Stx toxoids was also achieved in mice [29, 30].

We therefore hypothesized that passive (maternal) and active vaccination against Stx1 and Stx2 confers protection against the toxins’ immunomodulating effect and subsequently enables calves to actively mount a rapid immune response against STEC strains circulating in the respective cohort. In order to follow a novel approach to add on current vaccination strategies aiming at reducing STEC shedding by cattle, this study wanted to assess if active and passive immunization with Stx toxoid-based vaccines enables calves (i) to gain and produce Stx-neutralizing antibodies and (ii) to mount a more rapid and effective cellular immune response against STEC strains than unvaccinated controls in a respective cohort (iii) resulting in reduction of STEC shedding. As an essential antioxidant for maintaining the stability of biological membranes and the function of the immune system vitamin E (vit E) is considered to support adaptive and humoral immune responses [31–34]. Therefore, milk replacer fed to some animals deployed in the study was supplemented with vit E in higher amounts as usually included in commercially available products to support the effect of the vaccine.

## Materials and methods

### Generation of recombinant Shiga toxins and Shiga toxoids

Recombinant Stx (rStx1_WT_ and rStx2_WT_) and genetically inactivated recombinant Stx toxoids (rStx1_MUT_ and rStx2_MUT_) were previously generated and tested by Kerner et al. [24]. rStx1_MUT_ and rStx2_MUT_ preparation were adjusted separately with NaCl solution (0.89%) to 0.75 Mio verocytotoxic doses 50% (CD_50_) equivalents [24] each in 1.4 mL and frozen at −20 °C.

### Vaccination of cows for colostrum production

In October and November 2012, 14 cows (Deutsche Holstein) from the dairy herd at the experimental station, FLI Brunswick, served as donors for colostrum to be used in the subsequent year. Five cows had been vaccinated at 9 and 6 weeks before the calculated calving date. To this end, cows received by separate injection in the left and right *M. gluteus* 0.75 Mio CD_50_ equivalents of rStx1_MUT_ and of rStx2_MUT_ each in 1.4 mL NaCl solution (0.89%) freshly mixed with 0.6 mL aluminum hydroxide (Alu-Gel-S, Serva Electrophoresis GmbH, Heidelberg, Germany). Further 9 cows served as unvaccinated controls. Colostrum was collected from the first 3 milkings after parturition and screened for Stx-neutralizing antibodies by Vero cell neutralization assay (VNA). Pools of colostrum with high (pool VAC+; anti-Stx1 titer 51 001, anti-Stx2 titer 32 856; determined by VNA, see below) and with lower anti-Stx titer (pool VAC−; anti-Stx1 titer 11 005, anti-Stx2 titer 15 811) were collected, aliquoted and stored (−20 °C).

### Experimental design, housing and sampling of calves

In October and November 2013, a total of 48 male Holstein calves born in the dairy herd of FLI Brunswick were alternately assigned to groups of to be rStx_MUT_-vaccinated animals (VAC+) and placebo-treated animals (VAC−), as well as to vit E high (VitE_H_) and vit E moderate (VitE_M_) feeding groups. The treatments were arranged according to a two by two complete 2-factorial design and consequently resulted in four experimental groups: VAC+ VitE_H_ (*n* = 11), VAC− VitE_H_ (*n* = 11), VAC+ VitE_M_ (*n* = 13) and VAC− VitE_M_ (*n* = 13).

Directly after birth, calves were separated from dams. In the first 9 (± 1.5) days of life animals were kept separately in calf huts on straw. Thereafter calves were kept in 2 groups in accordance with vit E feeding assignment with straw bedding. From weaning until the end of the observation period, animals were housed in groups of different sizes irrespective of their group affiliation together with other bull calves of the same age, not included in the trial. Fecal samples were collected in weeks 3, 16, 26 and 55. Serum samples were collected before initial colostrum intake (pre-colostral), between 6 to 24 h after initial colostrum intake (post-colostral), as well as in weeks 3, 11, 16, 26, 55. For PBMC preparation 16 mL blood (week 16 and 26) were drawn from the jugular vein into tubes containing 4 mL 3.8% sodium citrate dihydrate (Sigma-Aldrich GmbH, Deisenhofen, Germany) solution. The vaccination protocol included a combination of passive and active vaccination with recombinant Shiga toxoids (rStx_MUT_). Directly after birth, VAC+ calves were fed 3 L of a colostrum pool previously collected from rStx_MUT_ vaccinated cows as described above while VAC− calves received colostrum from non-vaccinated cows. In the 5^th^ and 8^th^ week of life VAC+ animals were actively vaccinated with rStx1_MUT_ and rStx2_MUT_ as described above. VAC− animals were placebo-injected with NaCl solution and adjuvant. After feeding 3 L colostrum, calves were offered 875 g/day of milk replacer (MR) supplying 188 and 200 IU all-rac-α-tocopheryl acetate/kg dry matter (DM) in group VitE_M_ and VitE_H_, respectively, dissolved in 6 L of water split into two equal portions (bucket feed) daily until the 9^th^ (± 1.5) day of live. The vit E dose fed to VitE_M_ group was referred to as “moderate” as the vit E concentration supplied with the feed was above levels of international recommendations for calves of the respective age [35]. However, the dose used was equivalent to the degree of supplementation of milk replacer commercially distributed and used in modern livestock farming. From 10^th^ to 49^th^ day of life VitE_H_ calves were offered 840 g/day MR supplemented with 188 IU and 354 IU all-rac-α-tocopheryl acetate/kg DM in group VitE_M_and VitE_H_ by automatic calf feeders (Förster-Technik GmbH, Engern, Germany). Vit E fed groups split by vaccination took up comparable average amounts of all-rac-α-tocopheryl acetate (IU/day: VAC+ VitE_H_ 279.2; VAC− VitE_H_ 276.9; VAC+ VitE_M_ 152.2; VAC− VitE_M_ 154.7) throughout the entire period of performance acquisition. From the 9^th^ day calves received hay ad libitum and concentrate up to 2 kg/day in addition. Drinking water was available for ad libitum intake during the whole experiment. From the 50^th^ day until the end of observation period animals were fed conventionally.

### Vero cell cytotoxicity assay (VCA) and Vero cell neutralization assay (VNA)

The VCA was performed in 96-well microtiter plates (Nunc GmbH, Wiesbaden, Germany) using Vero cells (ATCC CRL 1587, LGC-Promochem GmbH, Wesel, Germany) to determine Vero cytotoxic doses 50% (CD_50_/mL) [36]. The VNA was used for the quantitation of the neutralizing activity in serum and colostrum against rStx1_WT_ or rStx2_WT_ as described previously [12]. Serum samples were tested at pre-dilutions of 1:30 and 1:90, colostrum samples at 1:300 and 1:900. At the pre-colostral sampling only a subset of animal’s sera (*n* = 23) was tested, at the other samplings all animals were included (*n* = 48). Neutralizing antibody (nAb) titers were determined by multiplying the relative cell activity with the dilution factor when the relative cell activity was > 30% (rStx1_WT_) or > 20% (rStx2_WT_). Samples with a relative cell activity below the detection limit (nAb rStx1 = 900; nAb rStx2 = 600) were given arbitrary nAb titer of half of the lowest detectable value.

### PBMC stimulation assay

Five *E. coli* and 2 *Listeria monocytogenes* strains isolated from dams of the experimental herd were cultivated in LB broth (lysogeny broth, Lennox), adjusted to 2 × 10^7^ colony forming units (cfu)/mL by optical density and heat inactivated (100 °C, 10 min). Aliquots of each lysate were tested for sterility on sheep blood agar (SIFIN diagnostics GmbH, Berlin, Germany) and stored at −20 °C until use. *E. coli* lysates were tested for cytotoxicity by VCA and for *stx*_1_ and *stx*_2_ by PCR. *E. coli* strain lysates were not cytotoxic except *E. coli* strain 2 (430 CD_50_/mL).

Peripheral blood mononuclear cells (PBMC) were isolated as described previously without erythrocytes lysis step [20]. PMBC were suspended in cell culture medium (RPMI 1640 supplemented with 10% fetal calf serum, 3 μM 2-mercaptoethanol, 100 IE/mL Penicillin, 2 mM l-glutamine) and seeded into Cellstar^®^ 24-well suspension plates (Greiner Bio-One GmbH, Frickenhausen, Germany) at 1 mL/well (2 × 10^6^ cells). Pure cell culture medium (MC) or cell culture medium supplemented with stimulants (lysates equivalent to final concentration (f.c.) of 1 × 10^5^ cfu/mL [17], Concanavalin A (ConA) f.c. 0.75 µg/mL, or rStx1_MUT_ or rStx2_MUT_ f.c. 200 CD_50_ equivalent [24] were added in 1 mL/well. Plates were incubated for 5 days at 5% CO_2_ and 37 °C.

**Figure 1:**
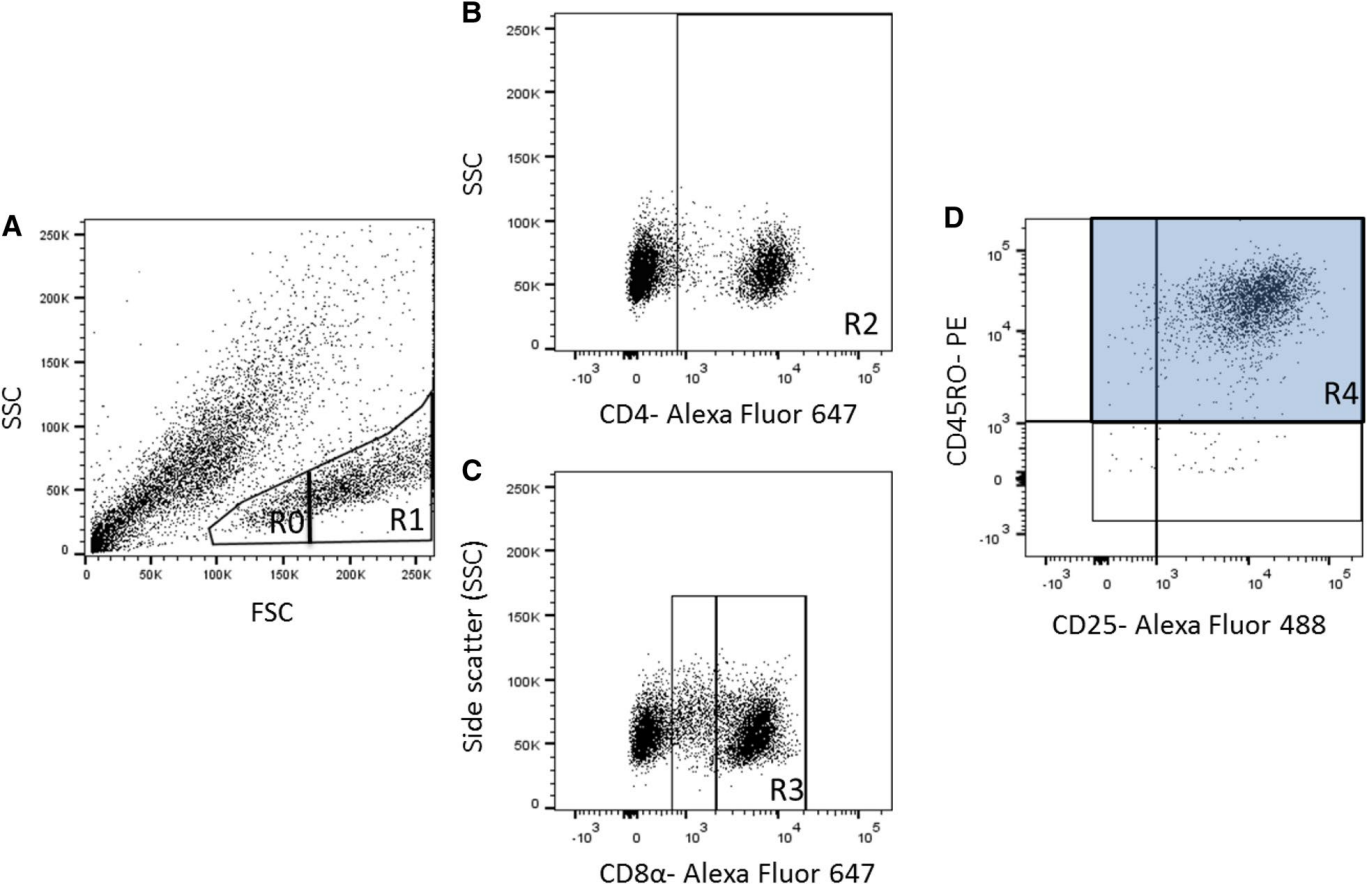
**Gating strategy used to quantify CD25 expression on inating vitro re-stimulated CD4**^**+**^**CD45RO**^**+**^ **and CD8α**^**hi**^**CD45RO**^**+**^** cells by flow cytometry.** Representative dot plots obtained with PBMC after 5 days of in vitro cultivation with Concanavalin A and subsequent tri-colour immunolabelling. **A** In a first analytical step, the non-blast cell (Region R0) and blast cell population (R1) were acquired for gating based on its forward versus side scatter (FSC/SSC) characteristics. **B** In samples labelled with anti-bovine CD4 antibodies, CD4^+^ cells within the blast cell population were defined by R2 as such that less than 2% of events were considered positive in the corresponding isotype control sample.** C** In samples labelled with anti-bovine CD8α antibodies, CD8α^hi^ cells were defined by R3, thereby excluding the majority of CD8α^lo^ cells.** D** Cells within R2 (CD4^+^) and R3 (CD8α^hi^) were displayed on CD45RO/CD25 dot plots and the geometric mean for detection of CD25 expression was assessed for all CD45RO^+^ within the CD4^+^ and CD8α^hi^, respectively, as defined by R4 (shaded area).

After 5-day incubation, supernatants were collected for further use (see below) and cells gently resuspended with washing buffer (PBS-Dulbecco without Ca and Mg, Biochrom supplemented with 0.01% sodium azide). Cell suspension was transferred to V-shape microtiter plates (Greiner Bio-One GmbH, Frickenhausen, Germany), incubated (10 min), and centrifuged (400 × *g*, 3 min, 4 °C). Supernatants were removed and cells resuspended with 30 µL of the primary monoclonal antibody dilution (anti-CD45RO clone IL-A116 [Bio-Rad AbD Serotec GmbH, Puchheim, Germany], 1:100). Cells were incubated (30 min, 4 °C, in the dark), washed twice, and resuspended with directly labelled antibodies (anti-CD4 Alexa Fluor 647 clone CC8; anti-CD8α Alexa Fluor 647 clone CC63; anti-CD25 FITC clone IL-A111 [Bio-Rad AbD Serotec GmbH, Puchheim, Germany], 1:200) and secondary antibody solutions (PE labelled α-rat IgG_2a_ ([QIAGEN Leipzig GmbH, Leipzig, Germany], 1:1000; for detection of anti-CD45RO) and incubated again. For isotype control, mouse IgG_2a_ (Alexa Fluor 647 [Bio-Rad AbD Serotec GmbH, Puchheim, Germany], 1:200) and mouse IgG_1_ (FITC [Bio-Rad AbD Serotec GmbH, Puchheim, Germany], 1:200) was used. Addition of PE-labelled α-rat IgG_2a_ to a control sample without anti-CD45RO was used to exclude non-specific binding of the indirectly labelled antibody. After washing, dead cells were quantified in the first set of experiments by incubating 50 µL cell suspension with 0.75 µL propidium iodide (Merck KGaA, Darmstadt, Germany) within MC. The proportions of dead cells were approx. 6% on average without group differences. At least 5000 cells with unaltered morphology (i.e. events which fell into the non-blast and blast region in the FSC vs. SSC plot) per sample, were assessed by use of a BD FACS Canto™ II analyzer (BD Biosciences, Heidelberg, Germany). CD4^+^CD45RO^+^ and CD8α^hi^CD45RO^+^ blast cells were analyzed for CD25 expression (FlowJo; Tree Star, Inc., San Carlos, CA, USA; see Figure 1 for the gating strategy). Results were expressed as percentage change compared to MC using the formula: (Geometric mean of fluorescence intensity [Geomean] stimulation—Geomean MC)/Geomean MC × 100%.

### IFN-γ ELISA of cell culture supernatants

PBMC supernatants were tested for IFN-γ protein by ID Screen^®^ ELISA test kit (ID.vet, Grabels) and evaluated following instructions provided by the manufacturer. Optical density (OD) was converted to S/P ratio following the equation: S/P ratio = [OD_stimulated sample_ – OD_medium control_l/OD_positive control_ × 100]. IFN-γ positive bovine activated plasma for positive control were obtained from the manufacturer.

### Estimation of quantity and quality of fecal STEC shedding

Approximately 10 g of fecal matter per animal was obtained directly from rectal lumen using sterile gloved fingers and stored at −80 °C. One gram was diluted in 9 mL sterile PBS buffer, homogenized, log_10_ diluted over five steps, and plated on Gassner agar (SIFIN diagnostics GmbH, Berlin, Germany). After incubation (18 h, 37 °C), representative agar plates were enumerated and cfu/g feces calculated. All coliform colonies from one 10^−1^ dilution plate were washed off with 1 mL LB broth supplemented with 30% glycerin, boiled 10 min at 100 °C, put on ice for 5 min and used as PCR template. *E. coli* strain EDL933 (kindly provided by Prof. R. Bauerfeind, Institute for Hygiene and Infectious Diseases of Animals, Justus Liebig University Giessen, Germany), positive for *stx*_1_ and *stx*_2_, served as a positive control. PCR primer pairs for detection of *stx*_1_ and *stx*_2_ were designed with reference to published sequence data [37]. The detection limit was determined to be 1 × 10^4^ cfu/g feces when two *stx*-negative fecal samples were spiked with 10^−1^ to 10^5^ cfu/g feces of viable *E. coli* EDL933 bacteria.

*Stx*-negative samples were excluded from further analysis. Colonies from *stx*-positive samples (*stx*_1_- and/or *stx*_2_-positive) were isolated by DNA–DNA colony hybridization as described by Geue et al. [13] with the following variations. DNA probes were labeled with digoxigenin with MP4/MP3 primers [37] using the PCR DIG Probe Synthesis Kit (Roche, Deutschland Holding GmbH, Grenzach Wyhlen, Germany) as specified by the manufacturer. Each *stx*-positive signal was assigned to a colony and up to 10 *stx*-positive colonies per blot were individually cultured in 200 µL LB broth, incubated (18 h, 37 °C), 30% glycerin added, and stored at −80 °C. The number of total *stx*-positive colonies on each blot was counted and cfu STEC/g feces calculated. Samples with *stx*-positive culture but no STEC detection by colony blot were given arbitrary STEC cfu/g feces of half of the dilution step used for blotting.

STEC isolates were characterized as to their possession of four virulence markers by multiplex PCR specific for *stx*_1_ and *stx*_2_ [37], *eae* [38]*, ehxA* (= *EHEC hlyA*) [39]. Profiles were defined by the presence of *eae* and/or *ehxA* in addition to *stx*_1_ and/or *stx*_2_. The numbers of different STEC profiles detectable in single fecal samples were recorded.

### Statistical analysis

Statistical analysis was done with “IBM SPSS statistics” (version 19, IBM Corporation, New York, USA) and XLSTAT-Pro (version 2015.1. Addinsoft, Paris, France). Fisher’s exact test was used to compare the PCR result of fecal *stx* status between the groups and to analyze if specific *stx* types detected in fecal cultures were differently distributed among trial groups. Friedman test was used to validate development of *E. coli* cfu and STEC cfu over time. Dunn’s test after a Kruskal–Wallis test and Mann–Whitney U test was performed for group comparison of *E. coli* cfu, STEC cfu, anti-Stx titer, INF-γ production and PBMC stimulation assay. Changes over time in anti-Stx titer were performed by Wilcoxon test. A value of *p* < 0.05 was considered statistically significant.

## Results

### Anti-Stx1 and anti-Stx2 titers in calf sera after passive and active immunization

**Figure 2:**
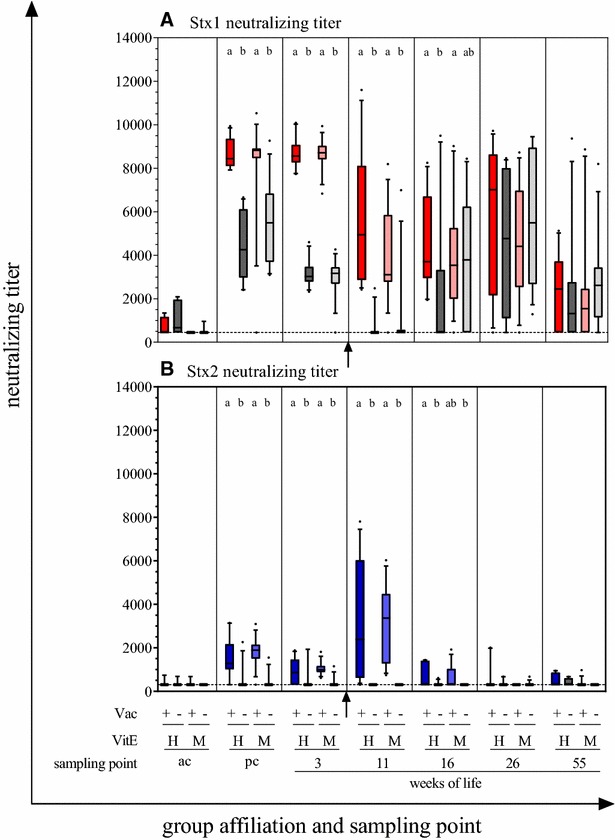
**Temporal pattern of Stx neutralizing titers in calves’ sera.** Antibody titers were determined before (ac = ante colostrum) and after (pc = post-colostral) colostrum intake as well as in the 3^rd^, 11^th^, 16^th^, 26^th^ and 55^th^ week of life. Animals were grouped according to their vaccination status (VAC+ = rStx_MUT_-vaccinated; VAC− = placebo control) and the supplementation of vitamin E (VitE_H_ = high supplementation; VitE_M_ = moderate supplementation). Arrows indicate active vaccination in the 5^th^ and 8^th^ week of life. **A** Temporal pattern of Stx1 neutralizing titers. A titer of 450 was attributed to those samples with a relative cell activity < 30% (detection limit, dashed line). **B** Temporal pattern of Stx2 neutralizing titers. A titer of 300 was attributed to those samples with a relative cell activity < 20% (detection limit, dashed line). Results of Vero cell neutralization assay expressed as box plots with 5–95% interquartile ranges. Different letters indicate significant differences between the groups at each time point separately based on Dunn’s test after a Kruskal–Wallis test.

Stx1- and Stx2-neutralizing antibodies (nStx1Ab/nStx2Ab) were detectable in pre-colostral sera of 2 of 9 tested VAC+ and 3 of 14 tested VAC− calves (Figure 2). After colostrum intake, nStx1Ab and nStx2Ab titers started to differ significantly between the VAC+ and the VAC− group. The nStx2Ab titer in 21 of 24 VAC− animals even remained below the detection limit. Regardless of vit E supplementation, the nStx1Ab and nStx2Ab serum titers continued to differ significantly between the VAC+ and the VAC− groups until 16^th^ week of life.

The nStx1Ab titer declined within the first 11 weeks in both groups (Figure 2A). In the VAC− group, nStx1Ab titers dropped below the detection limit in the 11^th^ week. In the 16^th^ week, most VAC− animals underwent a nStx1Ab seroconversion with titers peaking in week 26. VAC+ animals showed a similar trend with clearly detectable nStx1Ab titers throughout and a slight increase from week 16 to week 26. nStx1Ab titers declined in both groups from week 26 to week 55. The feeding with standard (“moderate”) or elevated (“high”) amounts of vit E had no influence on nStx1Ab titers, except within the VAC− group in the 16^th^ week. nStx1Ab titers had increased significantly to levels indistinguishable from titers of VAC+ animals in the VAC− VitE_M_ sub-group (9 of 13 animals seroconverted) but not in the VAC− VitE_H_ sub-group (9 of 11 seroconverted).

Independent of vit E supplementation, maternal nStx2Ab titers in VAC+ animals had already declined until week 3. Application of the vaccines between week 3 and 11 led to a clear increase in nStx2Ab titers, which only lasted until week 16, however. VAC− animals had very low to undetectable levels of nStx2Ab from week 11 onwards.

### IFN-γ production by PBMC after in vitro stimulation

**Figure 3:**
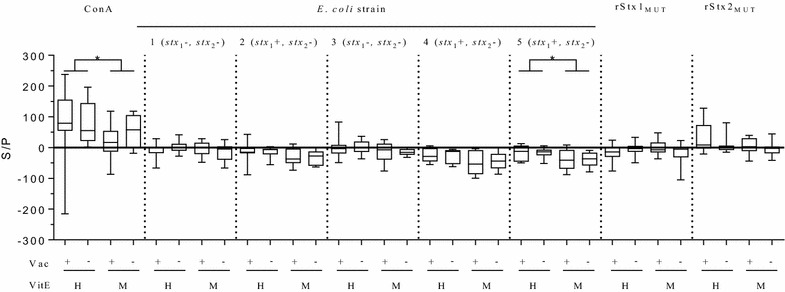
**IFN-γ production by PBMC after in vitro stimulation.** PBMC were isolated from calves in 16^th^ week of life, 8 weeks after active immunization with rStx_MUT_. Animals were grouped according to their vaccination status (VAC+ = rStx_MUT_-vaccinated; VAC− = placebo control) and the supplementation of vitamin E (VitE_H_ = high supplementation; VitE_M_ = moderate supplementation). PBMC were stimulated with lysates of 5 *E. coli* strains previously isolated at the farm and positive (*stx1* +) or negative (*stx* −) for *stx*. ELISA values obtained with cell culture supernatants after 24 h of stimulation are depicted in box plots (5–95% interquartile ranges) as percentages of positive controls (S/P). *Asterisk indicates statistical significance (*p* < 0.05) using Kruskal–Wallis test.

PBMC isolated from calves in the 16^th^ week of life secreted IFN-γ in higher amounts upon 24 h stimulation with ConA than PBMC in the medium control. Thereof, PBMC from VitE_H_ animals produced and secreted more IFN-γ than PBMC from VitE_M_ animals irrespective of rStx_MUT_ vaccination (Figure 3). Cultivation with lysates from *stx*-negative *E. coli* strains previously isolated from the farm, or with rStx1_MUT_ or rStx2_MUT_ did not induce significantly increased IFN-γ secretion by PBMC from animals of all groups. After cultivation with *stx*_1_-positive *E. coli* strain lysates, IFN-γ concentrations even tended to decrease in PBMC supernatants of the VitE_M_ fed animals compared to VitE_H_ fed animals. Differences reached statistical significance after stimulation with *E. coli* strain 5.

### CD25 expression on CD4^+^CD45RO^+^ and CD8α^hi^CD45RO^+^ cells after in vitro stimulation

**Figure 4:**
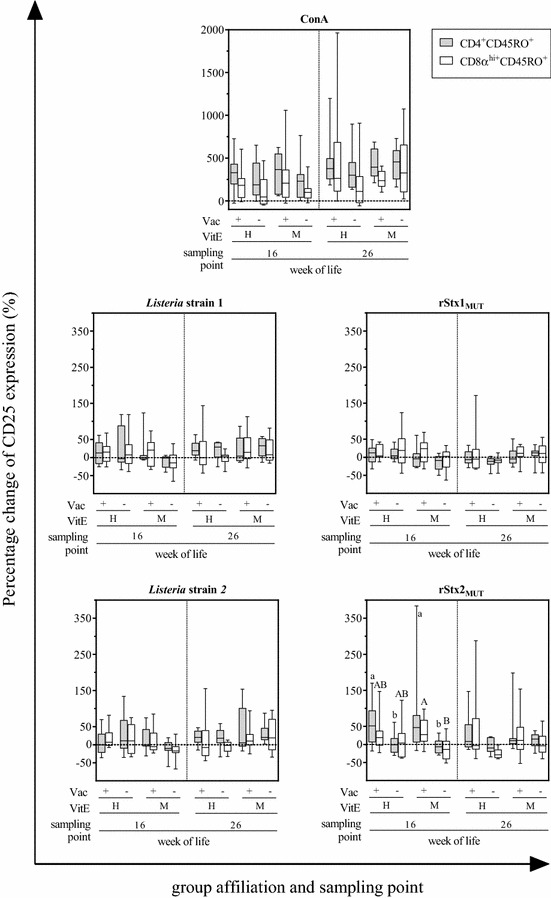
**Expression of CD25 on CD4 **^**+**^**CD45RO**^**+**^ **and CD8α**^**hi**^**CD45RO**^**+**^ **cells after cultivation with ConA, Shiga toxoids or** ***Listeria monocytogenes*** **lysates.** PBMC were isolated from calves in their 16^th^ and 26^th^ week of life, corresponding 8 and 18 weeks after active immunization with rStx_MUT_. Animals were grouped according to their vaccination status (VAC+ = rStx_MUT_-vaccinated; VAC− = placebo control) and the supplementation of vitamin E (VitE_H_ = high supplementation; VitE_M_ = moderate supplementation). Data obtained by flow cytometric analysis of the PBMC cultures after 5 days cultivation is depicted in box plots (5–95% interquartile ranges) as change of the geometric mean of fluorescence intensity of CD25 on CD4^+^CD45RO^+^ and CD8α^hi^CD45RO^+^ relative to unstimulated control cells (the latter values defined as 0, indicated by the dashed line). Different letters indicate significant differences between the groups in CD4^+^CD45RO^+^ (lower case letters) and CD8α^hi^CD45RO^+^ (capital letters) cells (*p* < 0.05, Kruskal–Wallis test with post hoc Dunn’s test).

**Figure 5:**
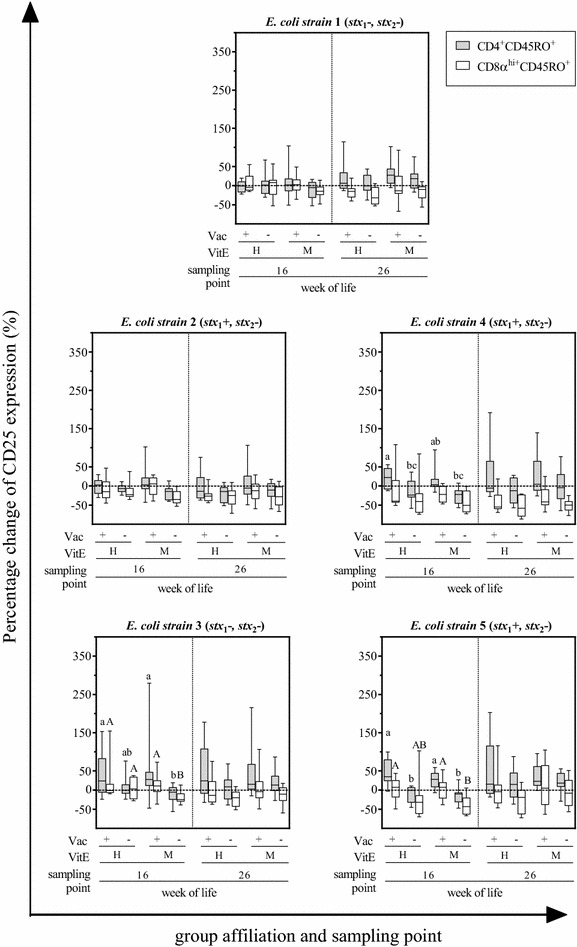
**Expression of CD25 on CD4**^**+**^**CD45RO**^**+**^ **and CD8α**^**hi**^**CD45RO**^**+**^ **cells after cultivation with** ***E. coli*** **lysates.** For details see legend to Figure 4.

CD4^+^CD45RO^+^and CD8α^hi^CD45RO^+^ cells responses to ConA, rStx1_MUT_ or *Listeria* strain lysates did not differ between calves of the VAC+ and the VAC− group (Figure 4). In contrast, cells obtained from VAC+ animals 8 weeks after vaccination (16^th^ week of life) responded to rStx2_MUT_ re-stimulation in vitro with an increase in the number of CD25 molecules on the cellular surface, whereas CD25 expression on CD4^+^CD45RO^+^and CD8α^hi^CD45RO^+^ of VAC− animals was indistinguishable from that of cells in the medium control. The effect of vaccination was significant for CD4^+^CD45RO^+^ from VitE_H_ and VitE_M_ animals, but for CD8α^hi^CD45RO^+^ cells in the VitE_M_ group only. Such an effect of rStx2_MUT_ re-stimulation on CD25 expression by CD8α^hi^CD45RO^+^ cells was also observed with PBMC obtained in the 26^th^ week but did not reach significant levels. Incubation with lysates of 1 out of 5 *E. coli* strains isolated from the herd (*E. coli* 1) did not affect CD25 expression (Figure 5). Even though group differences did not always reach significant levels, CD4^+^CD45RO^+^and CD8α^hi^CD45RO^+^ cells of VAC+ animals responded to lysates from the other 4 *E. coli* isolates with CD25 up-regulation, while respective cells from VAC− animals did not. Differences in responsiveness occurred in week 16, but not in week 26 and were more often significantly different in the VitE_M_ as in the VitE_H_ group.

### Fecal cultures and *stx*_1_-/*stx*_2_-PCR

Coliform colony-forming units per gram feces (cfu/g) in samples collected in the 3^rd^ week of life (median with 95% interquartile range: 3.3 × 10^6^ [1.3 × 10^6^–9.0 × 10^6^]) were significantly higher (*p* < 0.001) than in samples from the 16^th^ (1.1 × 10^5^ [4.1 × 10^4^–2.6 × 10^5^]), 26^th^ (1.4 × 10^5^ [7.7 × 10^4^–2.6 × 10^5^]), or 55^th^ (4.0 × 10^4^ [3.5 × 10^3^–8.0 × 10^4^]) week of life independent of group affiliation (Additional file 2).

In an attempt to unveil potential qualitative and quantitative changes in STEC shedding resulting from Stx toxoid vaccination, quantification of shedding as well as isolation and characterization of strains was conducted by applying an analytical workflow based on (i) detection of *stx* genes in the feces, (ii) *stx*-specific colony blotting, isolation and quantification of signal-positive colonies, and (iii) characterization of STEC isolates by multiplex PCR (see subsequent paragraph).

**Figure 6:**
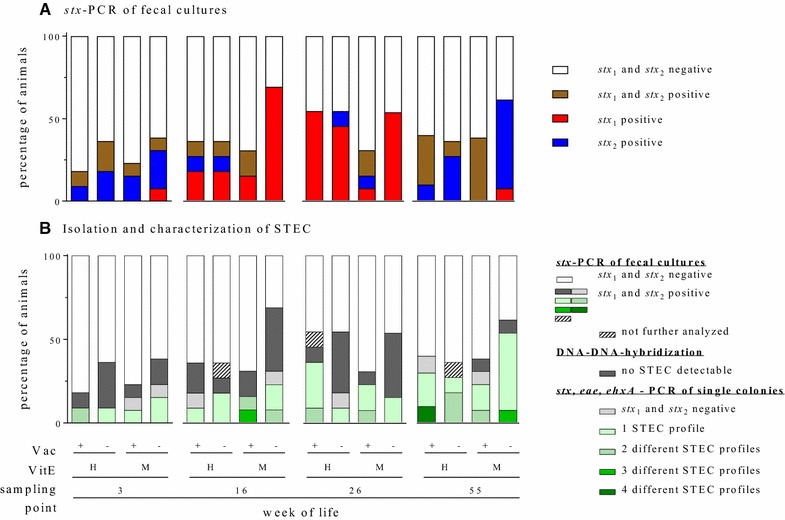
**Quantitative and qualitative assessment of fecal STEC shedding.** Animals were grouped according to their vaccination status (VAC+ = rStx_MUT_-vaccinated; VAC− = placebo control) and the supplementation of vitamin E (VitE_H_ = high supplementation; VitE_M_ = moderate supplementation). **A** Fecal cultures (n = 191) were screened for *stx*_1_/*stx*_2_ by PCR and grouped into *stx*-negative, *stx*_1_-positive, *stx*_2_-positive and *stx*_1_ plus *stx*_2_-positive. **B** Samples detected by *stx*-PCR of fecal cultures as *stx*_*1*_ and *stx*_*2*_ negative samples were not further analyzed (white part of the bar). Nylon membrane blots of agar plates of positively tested feces samples were hybridized with *stx*_1_/*stx*_2_ probes. Single colonies tested positive by hybridization (up to 10 per blot) were picked and further analyzed by PCR. A STEC profile is defined as single colony testing positive for *stx* (*stx*_*1*_ and/or *stx*_*2*_) plus facultative *eae* and/or *ehxA.* Different STEC profiles are illustrated with differently colored bars. Samples with no detectable *stx*_1_-/*stx*_2_-positive signal by hybridization (dark grey bars) and samples with single colonies positive detected by hybridization but negative signal by multiplex PCR (bright grey bars) are illustrated as such.

Taking into account all samples from VAC+ (*n* = 95) and VAC− calves (*n* = 96) over time, significantly (*p* = 0.040) less fecal samples collected from VAC+ animals were *stx*-positive by PCR than samples from VAC− animals (32/95 [33.7%] versus 47/96 [49.0%] samples in the VAC+ and the VAC− group, respectively). At each individual sampling point, less samples isolated from VAC+ animals were positive for *stx*-specific DNA than samples from VAC− animals, but those differences did not reach statistical significance (Figure 6A). The level of vit E supplementation did not influence the frequency of *stx*-positive fecal samples on its own. However, significant (*p* = 0.017) less samples collected from VAC+ VitE_M_ animals (16/52 [30.8%]) were *stx*-positive than from VAC− VitE_M_ animals (29/52 [55.8%]) over the whole observation period. In contrast, samples from VAC+ VitE_H_ (16 *stx*-positive of 43 [37.2%]) and from VAC− VitE_H_ (18/44 [40.9%]) animals did not differ significantly (*p* = 0.827). Three and two animals in the VAC+ and the VAC− group remained *stx*-negative, respectively, throughout the entire observation study. All other animals shed STEC, as deduced from a positive PCR result for *stx*_1_ or *stx*_2_, at least on one occasion. It cannot be ruled out that single PCR-positive fecal samples result from passaging of the bacteria rather than true infection. As a proxy of infection, 7 and 14 animals in the VAC+ and in the VAC− group, respectively, were found to be fecal-positive for at least one toxin type on at least two consecutive sampling times. Again, the VitE_H_ and VitE_M_ groups differed in their STEC shedding pattern. The number of animals that remained *stx*-negative were evenly distributed (VAC+ VitE_H_: 1/11, VAC− VitE_H_: 2/13; VAC+ VitE_M_: 1/11; VAC− VitE_M_: 1/13). However, 10 calves in the VAC− VitE_M_ group but only 5, 2 and 4 calves in the VAC+ VitE_H_, the VAC− VitE_H_ and the VAC+ VitE_M_ groups, respectively, were fecal-positive for *stx* on at least two consecutive sampling times (Additional file 1).

Considering *stx*-positive fecal samples only, such samples from VAC+ animals contained significantly (*p* = 0.003) more often *stx*_1_ plus *stx*_2_ (14/32; 43.8%) than samples from VAC− animals (6/47; 12.8%; Figure 6B). In turn, single *stx*_1_- or single *stx*_2_-positive samples were detected more often in VAC− (27/47 [51.1%] and 17/47 [36.2%], respectively) than in VAC+ (11/32 [34.4%] and 7/32 [21.9%], respectively) animals but differences did not reach statistical different levels (*p* ≥ 0.171). STEC shedding significantly varied with vit E supplementation also. A greater proportion of fecal samples from VAC+ VitE_M_ animals was *stx*_1_*/stx*_2_-positive (11/16; 68.8%) compared to VAC+ VitE_H_ (3/16; 18.8%; *p* = 0.011), VAC− VitE_H_ (4/28; 22.2%; *p* = 0.014), and VAC− VitE_M_ (2/29; 6.9%; *p* < 0.001). In contrast, significantly (*p* = 0.013) less fecal samples were single *stx*_1_-positive (3/16) in the VAC+ VitE_M_ group compared to the VAC− VitE_M_ (17/29; 18.8% versus 58.6%) group. For individual pattern of *stx*-positive fecal cultures see Additional file 1.

### Isolation and characterization of STEC strains

The percentage of *stx* PCR-positive fecal samples of which the subsequently conducted colony blotting did not yield a positive signal was different between the VAC+ (29.0%) and the VAC− groups (46.7%). STEC cfu per g of *stx* PCR-positive fecal samples did not differ (*p* = 0.216) between the trial groups over the entire observation period (Additional file 2). From a total of 40 fecal samples, that had at least one positive signal on the colony blot, 215 single colonies (VAC+ *n* = 108, VAC− *n* = 107 colonies) were analyzed to assess their virulence gene profile in order to assess possible implications of the vaccination for the composition and zoonotic potential of the STEC strains shed. As a result, isolates could be grouped into 12 virulence gene profiles based on the possession of *stx*_1_, *stx*_2_, *eae* or *ehxA*. Continuing with only one strain per virulence gene profile within a single sample, 64 individual STEC isolates were further analyzed (Table 1). More *stx*_1_–positive isolates were also positive for *eae* (59.1%) than *stx*_2_- and *stx*_1_/*stx*_2_-positive isolates (28.0 and 28.6%). The *ehxA* gene was detected in 77.3% of *stx*_1_-, 84.0% of *stx*_2_- and 28.6% of *stx*_1_-/*stx*_2_-positive isolates. Ten isolates lost the *stx* gene upon sub-culturing as they were originally positive by colony blot. The relative number of *stx*-blot positive samples harbouring more than one gene profile varied between VAC+ and VAC− animals (38.9 and 19.0%, respectively) (Figure 5B), indicating that vaccination had a qualitative impact on STEC shedding.

**Table 1:** Occurrence of individual STEC gene profiles in fecal samples

STEC gene profiles	Number of STEC profiles per group				
	VAC+ VitE_H_	VAC− VitE_H_	VAC+ VitE_M_	VAC− VitE_M_	Total
*stx*_1_	1	0	1	1	3
*stx*_1_ *, eae*	1	1	0	0	2
*stx*_1_ *, ehxA*	2	0	2	2	6
*stx*_1_ *, eae, ehxA*	3	1	3	4	11
*stx*_2_	0	0	0	2	2
*stx*_2_ *, eae*	0	0	1	1	2
*stx*_2_ *, ehxA*	3	3	5	5	16
*stx*_2_ *, eae, ehxA*	1	2	1	1	5
*stx*_1_ *, stx*_2_	1	1	1	1	4
*stx*_1_ *, stx*_2_ *, ehxA*	1	0	0	0	1
*stx*_1_ *, stx*_2_ *, eae, ehxA*	0	1	0	0	1
*stx*_1_ *, stx*_2_ *, ehxA*	1	0	0	0	1
*eae* ^a^	1	1	1	0	3
Negative^a^	1	3	1	2	7
Total	16	13	16	19	64

Fecal samples were taken over the entire observation period according to the group affiliation of the calves. Profiles were defined by the genes *stx*_*1*_, *stx*_*2*_, *eae* and *ehxA* and the numbers of different STEC profiles detectable in single fecal samples were recorded, whereby isolates with the same STEC gene profile within a single fecal sample were counted once.

^a^All isolates were initially *stx*-positive as their selection was based on the detection of *stx* by colony hybridization.

## Discussion

Up to now, experimental and licensed vaccines for reduction of STEC shedding (Econiche Corp, Belleville, Canada; Epitopix, Willmar, USA) were only partially successful and effects were mostly restricted to subpopulations of STEC, e.g. O157:H7/H– [14–16]. This proof-of-concept study provides first evidence that immunization with Stx toxoid-based vaccines under field-like conditions enables calves to actively mount a more effective immune response against STEC strains circulating in the cohort. Observed effects of rStx_MUT_ immunization on the incidence of *stx*-positive fecal samples were moderate compared to effects reportedly seen after immunization of cattle with STEC adhesins and subsequent homologous experimental challenge [14, 16]. However, the immune response primarily analysed herein was only indirectly achieved through natural exposure to a bacterial pathogen known to asymptomatically colonize the bovine intestine. Furthermore, vaccinated and control animals were kept together and participated in the same network of animal-to-animal and environmental-to-animal transitional events. The study did not include an immunological naïve control group, i.e. the colostrum pool administered to control calves was not free of anti-Stx antibodies. These confounding factors make the degree of differences in cellular immune responses and STEC shedding between animals groups highly plausible.

Colostral nStx1Ab and nStx2Ab were effectively transferred to calves with nStx1Ab titers clearly exceeding nStx2Ab titers in the VAC+ as well as the VAC− group similar to what was observed after natural Stx exposure and after rStx_MUT_ vaccination [12, 24, 40]. Active rStx_MUT_ immunization in week 5 and 8 did not result in a detectable increase of nStx1Ab in calves’ sera, different from calves vaccinated after vanishing of maternal antibodies [24]. High maternal titers may have impaired the success of the rStx1_MUT_ vaccination but a significant humoral immune response was achieved by rStx2_MUT_ immunization of calves with no or low serum nStx2Ab titers at the time of active vaccination. A modified vaccination scheme, consisting of maternal vaccination, prime vaccination of calves not earlier than week 11 and booster application in the second half of the first year of life might overcome these obstacles.

Applying the quantitation of CD25 expression on CD45RO^+^ lymphocytes for monitoring antigen-specific T cell responses in cattle, as suggested by Koo et al. [41], we found that peripheral CD4^+^CD45RO^+^ and CD8α^hi^CD45RO^+^ cells from VAC+ calves responded better to re-stimulation with STEC antigens. Eight weeks after active vaccination, PBMCs from VAC+ animals responded more pronounced to rStx2_MUT_ re-stimulation in vitro suggesting that vaccination directly induced an adaptive immune response against Stx2. The missing T cell responsiveness to rStx1_MUT_ in vitro may result from high maternal humoral protection at the time of active vaccination as discussed for the humoral response. Of note, VAC+ calves were capable of mounting an earlier adaptive immune response to *E. coli* antigens other than Stx1 as well. This became apparent by re-stimulation with *stx*_1_ +/*stx*_2_ − *E. coli* lysates in the 16^th^ week of life. The CD4^+^ subset analysed may have comprised different T_H_ subsets as well as T_reg_ cells. Bovine NK cells express CD45RO, CD8αα and CD8αβ [42] and are able to respond to bacterial molecular patterns without MHC restriction [43–45]. By gating on CD8α^hi^ cells, we aimed at excluding NK as well as γδT cells which both may express CD8α^lo^ [43, 46]. PBMC of the calves in the VAC+ and the VAC− group responded differently to some but not all bacterial lysates implying that the response detected was not due to an innate-like immune response but, in support of our hypothesis, indicative of an elevated adaptive cellular immune response to STEC strains circulating in the herd. Antibodies to STEC antigens may not be sufficient to protect cattle from STEC colonization [47]. Corbishley et al. showed by characterizing the epitopes recognized by CD4^+^ T-cells that STEC-infected calves develop a specific immunological response at the infection site [48]. In light of these findings, the relative importance of functionally different immune cell populations for the control of STEC colonization in cattle’s intestine urgently needs to be unveiled.

Hoffmann et al. showed that calves inoculated twice with *stx*-negative O157 strain, but not the calves inoculated with *stx*_2_-positive O157 strain, developed cellular immune responses against the latter strain [17]. Under less standardized field conditions in the current study, VAC+ animals also responded to one of the two *stx*-negative *E. coli* strains better than VAC− animals, implying that rStx_MUT_ vaccination enables calves to build an infection immunity towards other antigens that are common in STEC and other *E. coli* strains. In order to determine if this supportive effect expands to infections with other enteric bacterial agents, immune control of which heavily relies on cellular immune responses, we also re-stimulated PBMC of the calves with lysates of *Listeria* strains previously isolated on the experimental farm. A beneficial effect of Stx_MUT_ vaccination on the immune response against *Listeria monocytogenes* in the cohort of calves under study could not be confirmed. We cannot rule out that this resulted from lack of exposure as we did not monitor the *Listeria* infection status of the calves. Nevertheless, T-cells from VAC+ animals, which we consider to have been less exposed to Stx, tended to respond more vigorously to ConA in week 16. Stx inhibits the immunogenic effect of systemically administered antigen [49] and Kieckens et al. as well as Corbishley et al. showed that STEC colonization affect cattle’s general immune response [50, 51] suggesting that Stx impairs immune responses in cattle in a more general manner than currently recognized.

The vaccine deployed in this study contained rStx1_MUT_ and rStx2_MUT_, respectively, but is not devoid of antigens derived from the laboratory *E. coli* K-12 strain used for recombinant protein expression. The control calves were placebo injected with NaCl solution and adjuvant to exclude the adjuvant’s influence. Registered O157-vaccines (Econiche Corp, Belleville, Canada; Epitopix, Willmar, USA) contain proteins from the LEE locus and/or siderophore proteins as the main vaccine component. Both potentially protective STEC antigens are not included in the genome of the *E. coli* K-12 strain. Moreover, residual *E. coli* antigens other than rStx1_MUT_ and rStx2_MUT_ in the vaccine preparations are unlikely to have had an effect on the parameters assessed. Firstly, the general coliform flora in the calves was not affected by vaccination as no differences in the colony count were measured. Secondly, T-cells of VAC+ calves responded to rStx2_MUT_ containing vaccine preparations derived from the laboratory *E. coli* K-12 strain in vitro but not to the rStx1_MUT_ containing preparation strongly arguing against a general anti-*E. coli* immunity induced by the vaccination itself.

The hypothesis that high vit E supplementation stimulates the immune system and thereby supports the effect of rStx_MUT_ vaccination to reduce STEC shedding could not be substantiated. The higher vit E supplementation had no positive effect on CD25 expression by CD4^+^D45RO^+^ and CD8α^hi^CD45RO^+^ after stimulation with STEC and *E. coli* and rather a negative effect on STEC shedding in VAC+ calves. Nevertheless, 354 IU compared to 188 IU daily vit E supplementation via milk replacer exhibited effects on STEC shedding within the VAC+ group for reasons that have not been unveiled yet and interactive effects of vit E supply and rStx_MUT_ vaccination on, e.g., feed intake (N. Schmidt, T. Luhmann, L. Hüther, U. Meyer, S. A. Barth, L. Geue, C. Menge, J. Frahm, S. Dänicke, submitted for publication) have also to be taken into consideration.

Shiga toxoid immunization did not influence the absolute amount of STEC in fecal samples that still were *stx* PCR-positive. Moreover, our data indicate that samples from VAC+ calves tended to have higher STEC cfu in relation to total coliform counts. As the methodical workflow allowed isolation of STEC via colony blotting only from samples with STEC cfu/g feces maximally 2 log_10_ levels lower than coliform flora, we assume that rStx_MUT_ vaccination reduced the incidence of STEC primarily in animals with low numbers of STEC within the coliform flora rather than affecting the shedding of animals with relatively high levels of STEC shedding, referred to as supershedders [52].

Descriptive analysis of virulence gene profiles of STEC isolates showed that VAC+ animals had higher number of different STEC profiles per fecal sample and that STEC strains isolated from VAC+ animals were significantly more often both *stx*_1_ and *stx*_2_ positive than isolates from VAC−. The effect of rStx_MUT_ vaccination is mainly indirect and the success of the vaccination approach depends on sufficient exposure to a relevant number of bacteria of all strains implicated in STEC transmission dynamics in a respective cattle herd. In order to avoid selection of more virulent STEC strains by vaccination of cattle, the immunogenic capacity (dose) of the toxoids, the vaccine formulation, and the time and route of application have to be optimized and other virulence factors conserved among STEC strains like intimin [51], EspB [53] or associated with the colonization type of STEC strains [6] have to be included in the vaccine and/or the feed composition. Nevertheless, data presented here support our hypothesis that the immune modulatory effect of Stx should be considered for integration in strategies aiming at reducing STEC shedding in cattle.

## Additional files

**Additional file 1. Individual pattern of** ***stx*-positive fecal cultures in the** **3**^**rd**^, **16**^**th**^, **26**^**th**^ **and** **55**^**th**^ **week of life.** Animals were grouped according to their vaccination status (VAC+ = rStx_MUT_-vaccinated; VAC− = placebo control) and the supplementation of vitamin E (VitE_H_ = high supplementation; VitE_M_ = moderate supplementation). White boxed depict *stx*_*1*_-/*stx*_*2*_-fecal cultures analyzed by multiplex PCR. Detection of *stx*_*1*_ and *stx*_*2*_ is marked in gray and black, respectively. White cross-out boxes mark a gap in sampling.

**Additional file 2. Quantitative assessment of fecal** ***E. coli*** **and STEC shedding.** Fecal *E. coli* colony forming units (cfu) are shown with respect to the total feces sampling pool (Shiga toxin positive and negative feces) and Shiga toxin positive pool of each trial group at each sampling.

## Abbreviations

Stx: Shiga toxin
STEC: Shiga toxin-producing *Escherichia coli*
rStx1_MUT_/rStx2_MUT_: recombinant Shiga toxoids
EHEC: enterohemorrhagic *Escherichia coli*
Vitamin E: vit E
VNA: Vero cell neutralization assay
VAC+: rStx_MUT_-vaccinated animals
VAC−: placebo-treated animals
VitE_H_: vit E high feeding group
PBMC: peripheral blood mononuclear cells
VitE_M_: vit E moderate feeding group
MR: milk replacer
DM: dry matter
VCA: Vero cell cytotoxicity assay
nAb: neutralizing antibody
cfu: colony forming units
ConA: Concanavalin A
MC: medium control
nStx1Ab/nStx2Ab: Stx1-/Stx2-neutralizing antibodies

## References

[1] EFSA (European Food Safety Authority), ECDC (European Centre for Disease Prevention and Control) (2017). The European Union summary report on trends and sources of zoonoses, zoonotic agents and food‐borne outbreaks in 2016. EFS2.

[2] Krogfelt KA (1991). Bacterial adhesion: genetics, biogenesis, and role in pathogenesis of fimbrial adhesins of *Escherichia coli*. Rev Infect Dis.

[3] Jordan DM, Cornick N, Torres AG, Dean-Nystrom EA, Kaper JB, Moon HW (2004). Long polar fimbriae contribute to colonization by *Escherichia coli* O157:H7 in vivo. Infect Immun.

[4] Ackers ML, Mahon BE, Leahy E, Goode B, Damrow T, Hayes PS, Bibb WF, Rice DH, Barrett TJ, Hutwagner L, Griffin PM, Slutsker L (1998). An outbreak of *Escherichia coli* O157:H7 infections associated with leaf lettuce consumption. J Infect Dis.

[5] Barth S, Tscholshiew A, Menge C, Weiss R, Baljer G, Bauerfeind R (2007). Virulence and fitness gene patterns of Shiga toxin-encoding *Escherichia coli* isolated from pigs with edema disease or diarrhea in Germany. Berl Munch Tierarztl Wochenschr.

[6] Barth SA, Menge C, Eichhorn I, Semmler T, Wieler LH, Pickard D, Belka A, Berens C, Geue L (2016). The accessory genome of Shiga toxin-producing *Escherichia coli* defines a persistent colonization type in cattle. Appl Environ Microbiol.

[7] Hussein HS, Sakuma T (2005). Prevalence of shiga toxin-producing *Escherichia coli* in dairy cattle and their products. J Dairy Sci.

[8] Menrath A, Wieler LH, Heidemanns K, Semmler T, Fruth A, Kemper N (2010). Shiga toxin producing *Escherichia coli*: identification of non-O157:H7-Super-Shedding cows and related risk factors. Gut Pathog.

[9] Hamm K, Barth SA, Stalb S, Geue L, Liebler-Tenorio E, Teifke JP, Lange E, Tauscher K, Kotterba G, Bielaszewska M, Karch H, Menge C (2016). Experimental infection of calves with *Escherichia coli* O104:H4 outbreak strain. Sci Rep.

[10] Naylor SW, Gally DL, Low JC (2005). Enterohaemorrhagic *E. coli* in veterinary medicine. Int J Med Microbiol.

[11] Caprioli A, Morabito S, Brugere H, Oswald E (2005). Enterohaemorrhagic *Escherichia coli*: emerging issues on virulence and modes of transmission. Vet Res.

[12] Fröhlich J, Baljer G, Menge C (2009). Maternally and naturally acquired antibodies to Shiga toxins in a cohort of calves shedding Shiga-toxigenic *Escherichia coli*. Appl Environ Microbiol.

[13] Geue L, Segura-Alvarez M, Conraths FJ, Kuczius T, Bockemuhl J, Karch H, Gallien P (2002). A long-term study on the prevalence of shiga toxin-producing *Escherichia coli* (STEC) on four German cattle farms. Epidemiol Infect.

[14] Vande Walle K, Vanrompay D, Cox E (2013). Bovine innate and adaptive immune responses against *Escherichia coli* O157:H7 and vaccination strategies to reduce faecal shedding in ruminants. Vet Immunol Immunopathol.

[15] Callaway TR, Anderson RC, Edrington TS, Genovese KJ, Harvey RB, Poole TL, Nisbet DJ (2004). Recent pre-harvest supplementation strategies to reduce carriage and shedding of zoonotic enteric bacterial pathogens in food animals. Anim Health Res Rev.

[16] Snedeker KG, Campbell M, Sargeant JM (2012). A systematic review of vaccinations to reduce the shedding of *Escherichia coli* O157 in the faeces of domestic ruminants. Zoonoses Public Health.

[17] Hoffman MA, Menge C, Casey TA, Laegreid W, Bosworth BT, Dean-Nystrom EA (2006). Bovine immune response to Shiga-toxigenic *Escherichia coli* O157: H7. Clin Vaccine Immunol.

[18] Stamm I, Mohr M, Bridger PS, Schropfer E, Konig M, Stoffregen WC, Dean-Nystrom EA, Baljer G, Menge C (2008). Epithelial and mesenchymal cells in the bovine colonic mucosa differ in their responsiveness to *Escherichia coli* Shiga toxin 1. Infect Immun.

[19] Moussay E, Stamm I, Taubert A, Baljer G, Menge C (2006). *Escherichia coli* Shiga toxin 1 enhances il-4 transcripts in bovine ileal intraepithelial lymphocytes. Vet Immunol Immunopathol.

[20] Menge C, Wieler LH, Schlapp T, Baljer G (1999). Shiga toxin 1 from *Escherichia coli* blocks activation and proliferation of bovine lymphocyte subpopulations in vitro. Infect Immun.

[21] Menge C, Stamm I, van Diemen PM, Sopp P, Baljer G, Wallis TS, Stevens MP (2004). Phenotypic and functional characterization of intraepithelial lymphocytes in a bovine ligated intestinal loop model of enterohaemorrhagic *Escherichia coli* infection. J Med Microbiol.

[22] Menge C, Stamm I, Wuhrer M, Geyer R, Wieler LH, Baljer G (2001). Globotriaosylceramide (Gb(3)/CD77) is synthesized and surface expressed by bovine lymphocytes upon activation in vitro. Vet Immunol Immunopathol.

[23] Stamm I, Wuhrer M, Geyer R, Baljer G, Menge C (2002). Bovine lymphocytes express functional receptors for *Escherichia coli* Shiga toxin 1. Microb Pathog.

[24] Kerner K, Bridger PS, Kopf G, Frohlich J, Barth S, Willems H, Bauerfeind R, Baljer G, Menge C (2015). Evaluation of biological safety in vitro and immunogenicity in vivo of recombinant *Escherichia coli* Shiga toxoids as candidate vaccines in cattle. Vet Res.

[25] Hovde CJ, Calderwood SB, Mekalanos JJ, Collier RJ (1988). Evidence that glutamic acid 167 is an active-site residue of Shiga-like toxin I. Proc Natl Acad Sci U S A.

[26] Yamasaki S, Furutani M, Ito K, Igarashi K, Nishibuchi M, Takeda Y (1991). Importance of arginine at position 170 of the A subunit of Vero toxin 1 produced by enterohemorrhagic *Escherichia coli* for toxin activity. Microb Pathog.

[27] Oanh TKN, Nguyen VK, de Greve H, Goddeeris BM, Urban JF (2011). Protection of piglets against Edema disease by maternal immunization with Stx2e toxoid. Infect Immun.

[28] Makino S, Watarai M, Tabuchi H, Shirahata T, Furuoka H, Kobayashi Y, Takeda Y (2001). Genetically modified Shiga toxin 2e (Stx2e) producing *Escherichia coli* is a vaccine candidate for porcine edema disease. Microb Pathog.

[29] Ohmura-Hoshino M, Yamamoto M, Yuki Y, Takeda Y, Kiyono H (2004). Non-toxic Stx derivatives from *Escherichia coli* possess adjuvant activity for mucosal immunity. Vaccine.

[30] Ishikawa S, Kawahara K, Kagami Y, Isshiki Y, Kaneko A, Matsui H, Okada N, Danbara H (2003). Protection against Shiga toxin 1 challenge by immunization of mice with purified mutant Shiga toxin 1. Infect Immun.

[31] Reddy PG, Morrill JL, Frey RA (1987). Vitamin E requirements of dairy calves. J Dairy Sci.

[32] Reddy PG, Morrill JL, Minocha HC, Stevenson JS (1987). Vitamin E is immunostimulatory in calves. J Dairy Sci.

[33] Pekmezci D, Cakiroglu D (2009). Investigation of immunomodulatory effects of levamisole and vitamin E on Immunity and some blood parameters in newborn Jersey calves. Vet Res Commun.

[34] Samanta AK, Dass RS, Rawat M, Mishra RC, Mehra UR (2006). Effect of dietary vitamin E supplementation on serum α-tocopherol and immune status of crossbred calves. Asian-Aust J Anim Sci.

[35] National Research Council (2001). Nutrient requirements of dairy cattle: Seventh revised edition, 2001//Nutrient requirements of dairy cattle.

[36] Gentry MK, Dalrymple JM (1980). Quantitative microtiter cytotoxicity assay for Shigella toxin. J Clin Microbiol.

[37] Muller D, Greune L, Heusipp G, Karch H, Fruth A, Tschape H, Schmidt MA (2007). Identification of unconventional intestinal pathogenic *Escherichia coli* isolates expressing intermediate virulence factor profiles by using a novel single-step multiplex PCR. Appl Environ Microbiol.

[38] Franck SM, Bosworth BT, Moon HW (1998). Multiplex PCR for enterotoxigenic, attaching and effacing, and Shiga toxin-producing *Escherichia coli* strains from calves. J Clin Microbiol.

[39] Paton AW, Paton JC (1998). Detection and characterization of Shiga toxigenic *Escherichia coli* by using multiplex PCR assays for stx1, stx2, eaeA, enterohemorrhagic *E. coli* hlyA, rfbO111, and rfbO157. J Clin Microbiol.

[40] Pirro F, Wieler LH, Failing K, Bauerfeind R, Baljer G (1995). Neutralizing antibodies against Shiga-like toxins from *Escherichia coli* in colostra and sera of cattle. Vet Microbiol.

[41] Koo HC, Park YH, Hamilton MJ, Barrington GM, Davies CJ, Kim JB, Dahl JL, Waters WR, Davis WC (2004). Analysis of the immune response to *Mycobacterium avium* subsp. *paratuberculosis* in experimentally infected calves. Infect Immun.

[42] Graham EM, Thom ML, Howard CJ, Boysen P, Storset AK, Sopp P, Hope JC (2009). Natural killer cell number and phenotype in bovine peripheral blood is influenced by age. Vet Immunol Immunopathol.

[43] Endsley JJ, Endsley MA, Estes DM (2006). Bovine natural killer cells acquire cytotoxic/effector activity following activation with IL-12/15 and reduce *Mycobacterium bovis* BCG in infected macrophages. J Leukoc Biol.

[44] Hope JC, Sopp P, Howard CJ (2002). NK-like CD8(+) cells in immunologically naive neonatal calves that respond to dendritic cells infected with *Mycobacterium bovis* BCG. J Leukoc Biol.

[45] Olsen I, Boysen P, Kulberg S, Hope JC, Jungersen G, Storset AK (2005). Bovine NK cells can produce gamma interferon in response to the secreted mycobacterial proteins ESAT-6 and MPP14 but not in response to MPB70. Infect Immun.

[46] Menge C, Dean-Nystrom EA (2008). Dexamethasone depletes gammadelta T cells and alters the activation state and responsiveness of bovine peripheral blood lymphocyte subpopulations. J Dairy Sci.

[47] Johnson RP, Cray WC, Johnson ST (1996). Serum antibody responses of cattle following experimental infection with *Escherichia coli* O157:H7. Infect Immun.

[48] Corbishley A, Ahmad NI, Hughes K, Hutchings MR, McAteer SP, Connelley TK, Brown H, Gally DL, McNeilly TN (2014). Strain-dependent cellular immune responses in cattle following *Escherichia coli* O157:H7 colonization. Infect Immun.

[49] Chu A (2010) Immunomodulation by Shiga toxin 2. PhD Thesis, University of Saskatchewan http://hdl.handle.net/10388/etd-08252010-163545

[50] Kieckens E, Rybarczyk J, Li RW, Vanrompay D, Cox E (2016). Potential immunosuppressive effects of *Escherichia coli* O157:H7 experimental infection on the bovine host. BMC Genomics.

[51] Corbishley A, Connelley TK, Wolfson EB, Ballingall K, Beckett AE, Gally DL, McNeilly TN (2016). Identification of epitopes recognised by mucosal CD4(+) T-cell populations from cattle experimentally colonised with *Escherichia coli* O157:H7. Vet Res.

[52] Matthews L, Low JC, Gally DL, Pearce MC, Mellor DJ, Heesterbeek JAP, Chase-Topping M, Naylor SW, Shaw DJ, Reid SWJ, Gunn GJ, Woolhouse MEJ (2006). Heterogeneous shedding of *Escherichia coli* O157 in cattle and its implications for control. Proc Natl Acad Sci U S A.

[53] Vande Walle K, Yekta MA, Verdonck F, de Zutter L, Cox E (2011). Rectal inoculation of sheep with *E. coli* O157:H7 results in persistent infection in the absence of a protective immune response. Vet Microbiol.

