# Supplementary figures and images for: Decreased STEC shedding by cattle following passive and active vaccination based on recombinant Escherichia coli Shiga toxoids
Source: Vet Res. 2018 Mar 7;49:28. doi: 10.1186/s13567-018-0523-0 (PMC5842637; doi:10.1186/s13567-018-0523-0)

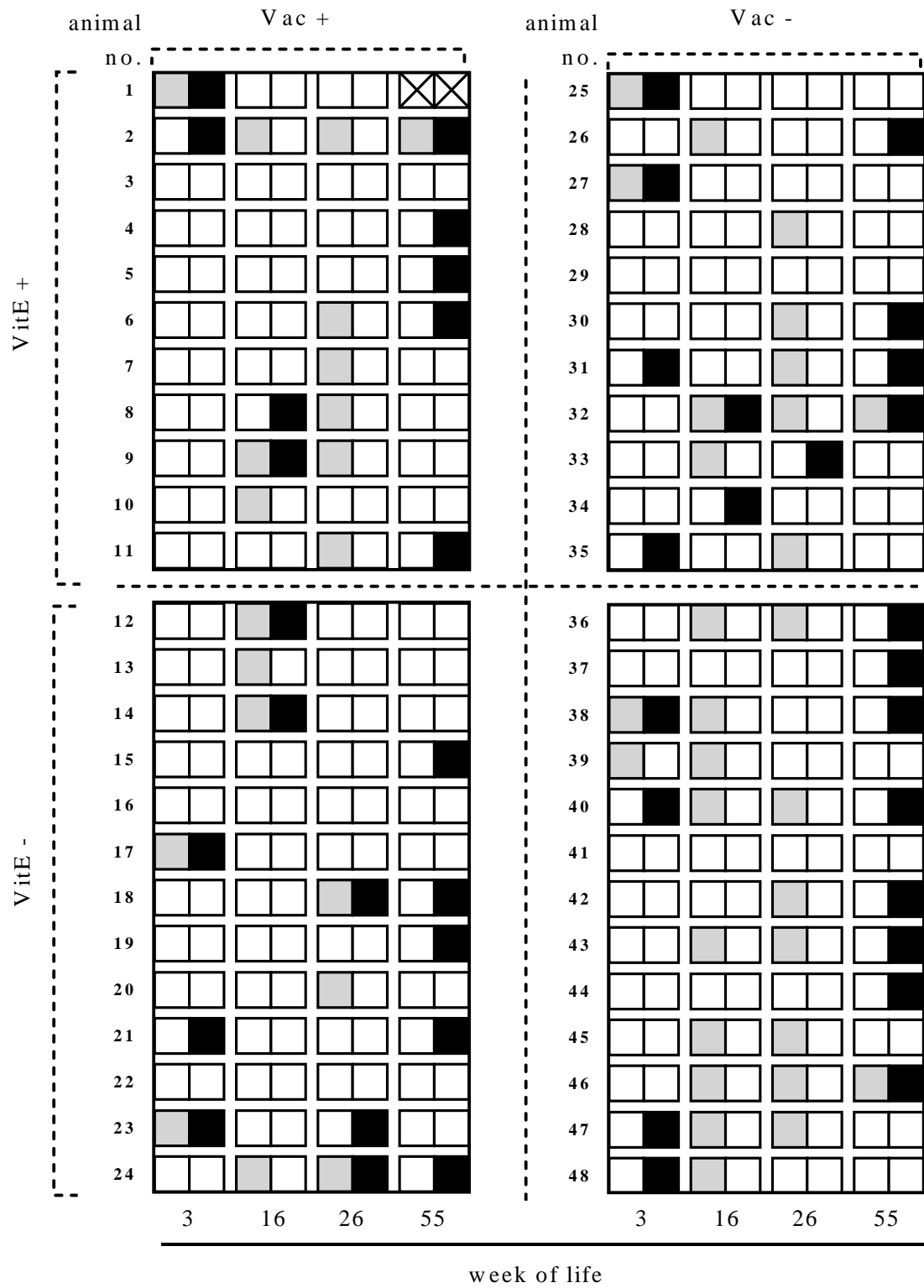

Supplement: Supplementary file 1 — Additional file 1. Individual pattern of stx-positive fecal cultures in the 3rd, 16th, 26th and 55th week of life. Animals were grouped according to their vaccination status (VAC+ = rStxMUT-vaccinated; VAC− = placebo control) and the supplementation of vitamin E (VitEH = high supplementation; VitEM = moderate supplementation). White boxed depict stx1-/stx2-fecal cultures analyzed by multiplex PCR. Detection of stx1 and stx2 is marked in gray and black, respectively. White cross-out boxes mark a gap in sampling. [file 13567_2018_523_MOESM1_ESM.pdf]
