# Supplementary material for: Decreased STEC shedding by cattle following passive and active vaccination based on recombinant Escherichia coli Shiga toxoids
Source: Vet Res. 2018 Mar 7;49:28. doi: 10.1186/s13567-018-0523-0 (PMC5842637; doi:10.1186/s13567-018-0523-0)
Supplement: Supplementary file 2 — Additional file 2. Quantitative assessment of fecal E. coli and STEC shedding. Fecal E. coli colony forming units (cfu) are shown with respect to the total feces sampling pool (Shiga toxin positive and negative feces) and Shiga toxin positive pool of each trial group at each sampling. [file 13567_2018_523_MOESM2_ESM.docx]

Additional file 2 Quantitative assessment of fecal *E. coli* and STEC shedding. Fecal *E. coli* colony forming units (cfu) are shown with respect to the total feces sampling pool (Shiga toxin positive and negative feces) and Shiga toxin positive pool of each trial group at each sampling.

| week of  life |  | *E. coli* cfu/g | | | | | | |  |  | | | |
| --- | --- | --- | --- | --- | --- | --- | --- | --- | --- | --- | --- | --- | --- |
|  |  | *stx*+ and *stx*- feces (n=182) | | |  | *stx*+ feces (n=75) | | |  | STEC cfu/g | | | |
|  | Group | Median | lower – upper  confidence limit (95 %) | | | Median | lower – upper confidence limit (95 %) | | | Median | | lower – upper  confidence limit (95 %) | |
| 3 | Vac VitE_H_ | 4.0 × 10^6^ | (6.7 × 10^5^ - | 2.2 × 10^7^) | | 2.9 × 10^6^ | (2.9 × 10^6^ - | 2.9 × 10^6^) | | 2.7 × 10^3^ | (300 - | | 5.0 × 10^3^) |
|  | Vac VitE_M_ | 2.7 × 10^6^ | (8.5 × 10^4^ - | 1.6 × 10^7^) | | 1.1 × 10^6^ | (1.1 × 10^5^ - | 9.0 × 10^6^) | | 2.8 × 10^3^ | (50 - | | 1.0 × 10^6^) |
|  | Mock VitE_H_ | 9.4 × 10^6^ | (1.1 × 10^6^ - | 1.6 × 10^7^) | | 7.8 × 10^6^ | (5.6 × 10^4^ - | 1.9 × 10^7^) | | 2.0 × 10^4^ | (500 - | | 2.0 × 10^5^) |
|  | Mock VitE_M_ | 2.8 × 10^6^ | (7.6 × 10^5^ - | 1.0 × 10^7^) | | 7.2 × 10^6^ | (5.6 × 10^4^ - | 1.6 × 10^7^) | | 5.0 × 10^3^ | (500 - | | 2.0 × 10^5^) |
| 16 | Vac VitE_H_ | 2.6 × 10^5^ | (6.1 × 10^3^ - | 9.5 × 10^5^) | | 5.5 × 10^5^ | (1.2 × 10^5^ - | 1.1 × 10^6^) | | 750 | (50 - | | 6.0 × 10^3^) |
|  | Vac VitE_M_ | 6.4 × 10^4^ | (2.6 × 10^4^ - | 1.4 × 10^6^) | | 5.4 × 10^4^ | (1.0 × 10^4^ - | 1.1 × 10^5^) | | 1.6 × 10^3^ | (500 - | | 1.4 × 10^4^) |
|  | Mock VitE_H_ | 5.4 × 10^4^ | (1.0 × 10^4^ - | 6.0 × 10^5^) | | 2.6 × 10^4^ | (7.6 × 10^3^ - | 1.4 × 10^6^) | | 430 | (50 - | | 2.7 × 10^3^) |
|  | Mock VitE_M_ | 6.5 × 10^4^ | (1.1 × 10^4^ - | 2.6 × 10^6^) | | 6.1 × 10^4^ | (6.1 × 10^3^ - | 2.1 × 10^5^) | | 50 | (50 - | | 1.6 × 10^3^) |
| 26 | Vac VitE_H_ | 7.3 × 10^4^ | (2.0 × 10^4^ - | 4.0 × 10^5^) | | 3.3 × 10^4^ | (2.0 × 10^4^ - | 3.9 × 10^5^) | | 1.0 × 10^3^ | (50 - | | 2.8 × 10^4^) |
|  | Vac VitE_M_ | 2.0 × 10^5^ | (8.5 × 10^4^ - | 5.5 × 10^5^) | | 8.4 × 10^4^ | (5.6 × 10^3^ - | 1.8 × 10^5^) | | 280 | (50 - | | 5.0 × 10^3^) |
|  | Mock VitE_H_ | 2.4 × 10^5^ | (7.7 × 10^4^ - | 3.7 × 10^5^) | | 1.5 × 10^5^ | (8.5 × 10^4^ - | 5.5 × 10^5^) | | 1.1 × 10^3^ | (50 - | | 4.0 × 10^3^) |
|  | Mock VitE_M_ | 9.8 × 10^4^ | (2.3 × 10^4^ - | 2.8 × 10^5^) | | 1.4 × 10^5^ | (9.8 × 10^3^ - | 3.5 × 10^5^) | | 50 | (5 - | | 3.0 × 10^4^) |
| 55 | Vac VitE_H_ | 4.7 × 10^4^ | (900 - | 9.8 × 10^4^) | | 2.9 × 10^4^ | (900 - | 3.0 × 10^5^) | | 3.4 × 10^3^ | (100 - | | 1.0 × 10^4^) |
|  | Vac VitE_M_ | 4.8 × 10^4^ | (1.8 × 10^3^ - | 9.6 × 10^4^) | | 1.3 × 10^4^ | (1.9 × 10^3^ - | 2.8 × 10^5^) | | 2.0 × 10^3^ | (300 - | | 3.0 × 10^3^) |
|  | Mock VitE_H_ | 1.2 × 10^5^ | (1.9 × 10^3^ - | 2.8 × 10^5^) | | 2.0 × 10^3^ | (1.8 × 10^3^ - | 8.2 × 10^4^) | | 400 | (50 - | | 1.1 × 10^3^) |
|  | Mock VitE_M_ | 3.5 × 10^3^ | (400 - | 8.2 × 10^4^) | | 5.7 × 10^3^ | (400 - | 8.2 × 10^4^) | | 400 | (50 - | | 2.0 × 10^3^) |
